# Bounds for the general sum-connectivity index of composite graphs

**DOI:** 10.1186/s13660-017-1350-y

**Published:** 2017-04-14

**Authors:** Shehnaz Akhter, Muhammad Imran, Zahid Raza

**Affiliations:** 1 School of Natural Sciences (SNS), National University of Sciences and Technology (NUST), Sector H-12, Islamabad, Pakistan; 2 Department of Mathematical Sciences, College of Science, United Arab Emirates University, P.O. Box 15551, Al Ain, United Arab Emirates; 3 Department of Mathematics, College of Sciences, University of Sharjah, Sharjah, United Arab Emirates

**Keywords:** 05C07, *R*-graphs, corona product, line graph, rooted product

## Abstract

The general sum-connectivity index is a molecular descriptor defined as $\chi_{\alpha}(X)=\sum_{xy\in E(X)}(d_{X}(x)+d_{X}(y))^{\alpha}$, where $d_{X}(x)$ denotes the degree of a vertex $x\in X$, and *α* is a real number. Let *X* be a graph; then let $R(X)$ be the graph obtained from *X* by adding a new vertex $x_{e}$ corresponding to each edge of *X* and joining $x_{e}$ to the end vertices of the corresponding edge $e\in E(X)$. In this paper we obtain the lower and upper bounds for the general sum-connectivity index of four types of graph operations involving *R*-graph. Additionally, we determine the bounds for the general sum-connectivity index of line graph $L(X)$ and rooted product of graphs.

## Introduction

Topological indices are useful tools for theoretical chemistry. A structural formula of a chemical compound is represented by a molecular graph. The atoms of the compounds and chemical bonds represent the vertices and edges of the molecular graphs, respectively. Topological indices related to their use in quantitative structure-activity (QSAR) and structure-property (QSPR) relationships are very interesting. In the QSAR/QSPR study, physico-chemical properties and topological indices such as the Wiener index, the Szeged index, the Randić index, the Zagreb indices and the ABC index are used to predict the bioactivity of the chemical compounds.

A single number that characterizes some properties corresponding to a molecular graph represents a topological index. There are many classes of topological indices, some of them are distance-based topological indices, degree-based topological indices and counting related polynomials and indices of graphs. All topological indices are useful in different fields, but degree-based topological indices play an important role in chemical graph theory and particularly in theoretical chemistry.

In this paper, we consider simple, connected and finite graphs. Let *X* be a graph with vertex set $V(X)$ and edge set $E(X)$. For $x\in V(X)$, $N_{X}(x)$ denotes the set of neighbors of *x*. The degree of a vertex $x\in V(X)$ is the number of vertices adjacent to *x* and represented by $d_{X}(x)=N_{X}(x)$. The numbers of vertices and number of edges in the graph *X* are represented by $n_{X}$ and $m_{X}$, respectively. The maximum and minimum vertex degree of *X* are denoted by $\bigtriangleup _{X}$ and $\delta_{X}$, respectively.

In the chemical and mathematical literature, several dozens of vertex-based graph invariants have been considered, in hundreds of published papers. For details see the books [1–3], and the surveys [4, 5].

The two oldest degree based molecular descriptors, called Zagreb indices [6], are defined as

$$ M_{1}(X)=\sum_{x\in V(X)} \bigl(d_{X}(x) \bigr)^{2},\quad\quad M_{2}(X)=\sum_{xy\in E(X)}d_{X}(x)d_{X}(y). $$

The general Randić index of *X* was proposed by Bollobás and Erdős [7], denoted by $R_{\alpha }(X)$ and defined as follows:

$$ R_{\alpha}(X)=\sum_{xy\in E(X)} \bigl(d(x)d(y) \bigr)^{\alpha}, $$

where *α* is a real number. Then $R_{-\frac{1}{2}}$ is the classical Randić index proposed by Randić [8] in 1975. Recently, a closely related topological index to the Randić index, called the sum-connectivity index [9], denoted by $\chi(X)$, is as follows:

$$ \chi(X)=\sum_{xy\in E(X)} \bigl(d_{X}(x)+d_{X}(y) \bigr)^{-\frac{1}{2}}. $$

Zhou and Trinajstić [10] introduced the general sum-connectivity index, denoted by $\chi_{\alpha}(X)$ and defined as follows:

1.1 $$ \chi_{\alpha}(X)=\sum_{xy\in E(X)} \bigl(d_{X}(x)+d_{X}(y) \bigr)^{\alpha}, $$

where *α* is a real number. Then $\chi_{-\frac{1}{2}}$ is the sum-connectivity index. Su and Xu [11] introduced a new topological index, the general sum-connectivity co-index, denoted by $\overline{\chi_{\alpha}}$ and is defined as follows:

$$ \overline{\chi_{\alpha}}(X)=\sum_{xy\notin E(X)} \bigl(d_{X}(x)+d_{X}(y) \bigr)^{\alpha}, $$

where *α* is a real number. Researchers introduced many graph operations such as the cartesian product, join of graphs, line graphs, the corona product, the edge corona product, the subdivision-vertex join, the subdivision edge join, the neighborhood corona, the subdivision vertex neighborhood corona and the subdivision edge neighborhood corona. Much work has been done related to these graph operations. Lan and Zhou [12] defined four new graph operations based on *R*-graphs and determined the adjacency (respectively, Laplacian and singles Laplacian) spectra of these graph operations. Godsil and McKay [13] introduced a new graph operation (rooted product) and then found its spectrum. Recently, Azari *et al.* [14] determined the Zagreb indices of chemical graphs that are constructed by a rooted product. Khalifeh *et al.* [15] gave the exact formulas for the Zagreb indices of several graph operations. For a detailed study of the topological indices of graph operations, we refer to [16–27].

## Methods

In this paper, we obtain the lower and upper bounds for the general sum-connectivity index of four types of graph operations involving the *R*-graph. Additionally, we determine the bounds for the general sum-connectivity index of line graph $L(X)$ and the rooted product of graphs by using graph-theoretic tools and mathematical inequalities.

## Results and discussion

In this section, we derive some bounds on the general sum-connectivity index of several graph operations such as *R*-graphs, line graphs and the rooted product. Let *X* and *Y* be two simple connected graphs whose vertex sets are disjoint. For each $x\in V(X)$ and $y\in V(Y)$, we have

3.1 $$\begin{aligned} \begin{gathered} \bigtriangleup_{X}\geq d_{X}(x), \quad\quad \delta_{X}\leq d_{X}(x), \\ \bigtriangleup_{Y}\geq d_{Y}(y),\quad\quad \delta_{Y} \leq d_{Y}(y). \end{gathered} \end{aligned}$$

The equality holds if and only if *X* and *Y* are regular graphs.

### *R*-graphs

Let *X* be a graph; then $R(X)$ is the graph obtained from *X* by adding a new vertex $x_{e}$ corresponding to each edge of *X* and joining $x_{e}$ to the end vertices of the corresponding edge $e\in E(X)$. Let $V(R(X))=V(X)\cup I(X)$, where $I(X)=V(R(X))\setminus V(X)$ is the set of newly added vertices.

The corona product of two graphs *X* and *Y*, denoted by $X\bullet Y$, is a graph obtained by taking one copy of graph *X* and $n_{X}$ copies of graph *Y* and joining the vertex *X*, that is, on the *i*th position in *X* to every vertex in *i*th copy of *Y*. The order and size of $X\bullet Y$ are $n_{X}(1+n_{Y})$ and $m_{X}+n_{X}m_{Y}+n_{X}n_{Y}$, respectively. The degree of a vertex $x\in V(X\bullet Y)$ is given by

3.2 $$ d_{X\bullet Y}(x)= \textstyle\begin{cases} d_{X}(x)+n_{Y} &\mbox{if }x\in V(X),\\ d_{Y}(x)+1 &\mbox{if }x\in V(Y). \end{cases} $$

Let *X* and *Y* be two connected and vertex-disjoint graphs. The *R*-vertex corona product of $R(X)$ and *Y*, denoted by $R(X)\odot Y$, is a graph obtained from one copy of vertex-disjoint graph $R(X)$ and $n_{X}$ copies of *Y* and joining a vertex of $V(X)$, that is, on the *i*th position in $R(X)$ to every vertex in the *i*th copy of *Y*. The graph $R(X)\odot Y$ has a number of $n_{X}+m_{X}+n_{X}n_{Y}$ vertices and $3m_{X}+n_{X}m_{Y}+n_{X}n_{Y}$ edges. The degree of a vertex $x\in V(R(X)\odot Y)$ is given by

3.3 $$ d_{R(X)\odot Y}(x)= \textstyle\begin{cases} 2d_{X}(x)+n_{Y} &\mbox{if }x\in V(X), \\ 2 &\mbox{if }x\in I(X),\\ d_{Y}(x)+1 &\mbox{if }x\in V(Y). \end{cases} $$

The *R*-edge corona product of $R(X)$ and *Y*, denoted by $R(X)\ominus Y$, is a graph obtained from one copy of vertex-disjoint graph $R(X)$ and $m_{X}$ copies of *Y* and joining a vertex of $I(X)$, that is, on *i*th position in $R(X)$ to every vertex in the *i*th copy of *Y*. The graph $R(X)\ominus Y$ has $n_{X}+m_{X}+m_{X}n_{Y}$ number of vertices and $3m_{X}+m_{X}m_{Y}+m_{X}n_{Y}$ number of edges. The degree of a vertex $x\in V(R(X)\ominus Y)$ is given by

3.4 $$ d_{R(X)\ominus Y}(x)= \textstyle\begin{cases} 2d_{X}(x) &\mbox{if }x\in V(X), \\ 2+n_{Y} &\mbox{if }x\in I(X),\\ d_{Y}(x)+1 &\mbox{if }x\in V(Y). \end{cases} $$

The *R*-vertex neighborhood corona product of $R(X)$ and *Y*, denoted by $R(X)\boxdot Y$, is a graph obtained from one copy of vertex-disjoint graph $R(X)$ and $n_{X}$ copies of *Y* and joining the neighbors of a vertex of *X* in $R(X)$, that is, on the *i*th position in $R(X)$ to every vertex in the *i*th copy of *Y*. The graph $R(X)\boxdot Y$ has $n_{X}+m_{X}+n_{X}n_{Y}$ vertices and $3m_{X}+n_{X}m_{Y}+4m_{X}n_{Y}$ edges. The degrees of vertices of $R(X)\boxdot Y$ are given by

3.5 $$ \begin{gathered} d_{R(X)\boxdot Y}(x)=d_{X}(x) (2+n_{Y}) \quad\mbox{if }x\in V(X), \\ d_{R(X)\boxdot Y}(x)=2(n_{Y}+1) \quad\mbox{if }x\in I(X), \\ d_{R(X)\boxdot Y}(y)=d_{Y}(y)+2d_{X}(x) \quad\mbox{if }y \in V(Y), x\in V(X). \end{gathered} $$

In the last expression, $y\in V(Y)$ is the vertex in *i*th copy of *Y* corresponding to *i*th vertex $x\in V(X)$ in $R(X)$.

The *R*-edge neighborhood corona product of $R(X)$ and *Y*, denoted by $R(X)\boxminus Y$, is a graph obtained from one copy of vertex-disjoint graph $R(X)$ and $m_{X}$ copies of *Y* and joining the neighbors of a vertex of $I(X)$ in $R(X)$, that is, on the *i*th position in $R(X)$ to every vertex in the *i*th copy of *Y*. The graph $R(X)\boxminus Y$ has $n_{X}+m_{X}+m_{X}n_{Y}$ vertices and $3m_{X}+m_{X}m_{Y}+2m_{X}n_{Y}$ edges. The degree of a vertex $x\in V(R(X)\boxminus Y)$ is given by

3.6 $$ d_{R(X)\boxminus Y}(x)= \textstyle\begin{cases} d_{X}(x)(2+n_{Y}) &\mbox{if }x\in V(X), \\ 2 &\mbox{if }x\in I(X),\\ d_{Y}(x)+2 &\mbox{if }x\in V(Y). \end{cases} $$

In the following theorem, we compute the bounds on the general sum-connectivity index of *R*-vertex corona product of $R(X)$ and *Y*.

#### Theorem 3.1

*Let* $\alpha<0$. *Then the bounds for the general sum*-*connectivity index of* $R(X) \odot Y$ *are given by*

$$\begin{aligned}& \begin{aligned} \chi_{\alpha} \bigl(R(X)\odot Y \bigr) & \geq 2^{\alpha}m_{X}(2\bigtriangleup _{X}+n_{Y})^{\alpha}+2m_{X}(2 \bigtriangleup_{X}+n_{Y}+2)^{\alpha} +2^{\alpha}n_{X}m_{Y}(\bigtriangleup_{Y}+1)^{\alpha} \\ &\quad{} +n_{X}n_{Y}(2\bigtriangleup_{X}+ \bigtriangleup_{Y}+n_{Y}+1)^{\alpha}, \end{aligned} \\& \begin{aligned} \chi_{\alpha} \bigl(R(X)\odot Y \bigr)&\leq2^{\alpha}m_{X}(2 \delta _{X}+n_{Y})^{\alpha}+2m_{X}(2 \delta_{X}+n_{Y}+2)^{\alpha}+2^{\alpha} n_{X}m_{Y}(\delta_{Y}+1)^{\alpha} \\ &\quad{} +n_{X}n_{Y}(2\delta_{X}+\delta_{Y}+n_{Y}+1)^{\alpha}. \end{aligned} \end{aligned}$$

*The equality holds if and only if* *X* *and* *Y* *are regular graphs*.

#### Proof

Using (3.1) and (3.3) in equation (1.1), we get

3.7 $$\begin{aligned} &\chi_{\alpha} \bigl(R(X)\odot Y \bigr) \\ &\quad =\sum_{xy\in E(R(X)} \bigl(d_{R(X)}(x)+d_{R(X)}(y) \bigr)^{\alpha}+n_{X}\sum_{xy\in E(Y)} \bigl(d_{Y}(x)+d_{Y}(y) \bigr)^{\alpha} \\ &\quad \quad{} +\sum_{x\in V(R(X))}\sum_{y\in V(Y)} \bigl(d_{R(X)}(x)+d_{Y}(y) \bigr)^{\alpha} \\ &\quad =\sum_{\substack{xy\in E(R(X)), \\x,y\in V(X)}} \bigl(2d_{X}(x)+2d_{Y}(y)+2n_{Y} \bigr)^{\alpha}+\sum_{\substack {xy\in E(R(X)), \\x\in V(X),y\in I(X)}} \bigl(2d_{X}(x)+n_{Y}+2 \bigr)^{\alpha} \\ &\quad \quad{} +n_{X}\sum_{xy\in E(Y)} \bigl(d_{Y}(x)+d_{Y}(y)+2 \bigr)^{\alpha}+\sum_{\substack{x\in V(R(X)) \\x\in V(X)}}\sum _{y\in V(Y)} \bigl(2d_{X}(x)+n_{Y}+d_{Y}(y)+1 \bigr)^{\alpha} \\ &\quad \geq2^{\alpha}m_{X}(2\bigtriangleup_{X}+n_{Y})^{\alpha }+2m_{X}(2 \bigtriangleup_{X}+n_{Y}+2)^{\alpha} +2^{\alpha}n_{X}m_{Y}(\bigtriangleup_{Y}+1)^{\alpha} \\ &\quad \quad{} +n_{X}n_{Y}(2\bigtriangleup_{X}+ \bigtriangleup_{Y}+n_{Y}+1)^{\alpha}. \end{aligned}$$

One can analogously compute the following:

3.8 $$ \begin{aligned}[b] \chi_{\alpha} \bigl(R(X)\odot Y \bigr)&\leq2^{\alpha}m_{X}(2\delta _{X}+n_{Y})^{\alpha}+2m_{X}(2 \delta_{X}+n_{Y}+2)^{\alpha}+ 2^{\alpha}n_{X}m_{Y}( \delta_{Y}+1)^{\alpha} \\ &\quad{} +n_{X}n_{Y}(2\delta_{X}+\delta_{Y}+n_{Y}+1)^{\alpha}. \end{aligned} $$

The equality in (3.7) and (3.8) obviously holds if and only if *X* and *Y* are regular graphs. This completes the proof. □

We compute the bounds on the general sum-connectivity index for the *R*-edge corona product of $R(X)$ and *Y* in the following theorem.

#### Theorem 3.2

*Let* $\alpha<0$. *Then the bounds for the general sum*-*connectivity index of* $R(X) \ominus Y$ *are given by*

$$\begin{aligned}& \begin{aligned} \chi_{\alpha} \bigl(R(X)\ominus Y \bigr) &\geq4^{\alpha}m_{X}\bigtriangleup _{X}^{\alpha}+2m_{X}(2 \bigtriangleup_{X}+n_{Y}+2)^{\alpha}+ 2^{\alpha}m_{X}m_{Y}(\bigtriangleup_{Y}+1)^{\alpha } \\ &\quad{} +m_{X}n_{Y}( \bigtriangleup_{Y}+n_{Y}+3)^{\alpha}, \end{aligned} \\& \begin{aligned} \chi_{\alpha} \bigl(R(X)\ominus Y \bigr)&\leq4^{\alpha}m_{X} \delta_{X}^{\alpha }+2m_{X}(2\delta_{X}+n_{Y}+2)^{\alpha}+2^{\alpha}m_{X}m_{Y} (\delta_{Y}+1)^{\alpha} \\ &\quad{} +m_{X}n_{Y}( \delta_{Y}+n_{Y}+3)^{\alpha}. \end{aligned} \end{aligned}$$

*The equality holds if and only if* *X* *and* *Y* *are regular graphs*.

#### Proof

Using (3.1) and (3.4) in equation (1.1), we get

3.9 $$\begin{aligned} &\chi_{\alpha} \bigl(R(X)\ominus Y \bigr) \\ &\quad =\sum_{xy\in E(R(X)} \bigl(d_{R(X)}(x)+d_{R(X)}(y) \bigr)^{\alpha}+m_{X}\sum_{xy\in E(Y)} \bigl(d_{Y}(x)+d_{Y}(y) \bigr)^{\alpha} \\ &\quad \quad{} +\sum_{x\in V(R(X))}\sum_{y\in V(Y)} \bigl(d_{R(X)}(x)+d_{Y}(y) \bigr)^{\alpha} \\ &\quad =\sum_{\substack{xy\in E(R(X)), \\x,y\in V(X)}} \bigl(2d_{X}(x)+2d_{Y}(y) \bigr)^{\alpha}+\sum_{\substack{xy\in E(R(X)), \\x\in V(X),y\in I(X)}} \bigl(2d_{X}(x)+n_{Y}+2 \bigr)^{\alpha} \\ &\quad \quad{} +m_{X}\sum_{xy\in E(Y)} \bigl(d_{Y}(x)+d_{Y}(y)+2 \bigr)^{\alpha}+\sum_{\substack{x\in V(R(X)) \\[-1pt]x\in I(X)}} \sum _{y\in V(Y)} \bigl(n_{Y}+2+d_{Y}(y)+1 \bigr)^{\alpha} \\ &\quad \geq4^{\alpha}m_{X}\bigtriangleup_{X}^{\alpha }+2m_{X}(2 \bigtriangleup_{X}+n_{Y}+2)^{\alpha}+2^{\alpha} m_{X}m_{Y}(\bigtriangleup_{Y}+1)^{\alpha} \\ &\quad\quad{} +m_{X}n_{Y}( \bigtriangleup _{Y}+n_{Y}+3)^{\alpha}. \end{aligned}$$

Similarly, we can show that

3.10 $$ \begin{aligned}[b] \chi_{\alpha} \bigl(R(X)\ominus Y \bigr) &\leq4^{\alpha}m_{X} \delta_{X}^{\alpha }+2m_{X}(2 \delta_{X}+n_{Y}+2)^{\alpha}+2^{\alpha} m_{X}m_{Y}(\delta_{Y}+1)^{\alpha} \\ &\quad{} +m_{X}n_{Y}(\delta_{Y}+n_{Y}+3)^{\alpha}. \end{aligned} $$

The equality in (3.9) and (3.10) obviously holds if and only if *X* and *Y* are regular graphs. This completes the proof. □

In the following theorem, we calculate bounds on the general sum-connectivity index for the *R*-vertex neighborhood corona product of $R(X)$ and *Y*.

#### Theorem 3.3

*Let* $\alpha<0$. *Then the bounds for the general sum*-*connectivity index of* $R(X)\boxdot Y$ *are given by*

$$\begin{aligned}& \begin{aligned} \chi_{\alpha} \bigl(R(X)\boxdot Y \bigr)& \geq2^{\alpha}m_{X}(n_{Y}+2)^{\alpha } \bigtriangleup_{X}^{\alpha}+2m_{X} \bigl(n_{Y}( \bigtriangleup_{X}+2) +2(\bigtriangleup_{Y}+1) \bigr)^{\alpha} \\ &\quad{} +2^{\alpha}n_{X}m_{Y}( \bigtriangleup_{Y}+2\bigtriangleup _{X})^{\alpha} \\ &\quad{} +n_{Y}\sum_{\substack{x\in V(X),\\[-1pt]w_{i}\in N_{X}(x), w_{i}\in V(X)}} \bigl( \bigtriangleup_{X}(n_{Y}+2)+\bigtriangleup_{Y}+2 \bigtriangleup _{X} \bigr)^{\alpha} \\ &\quad{} +n_{Y}\sum_{\substack{x\in V(X),\\[-1pt]w_{i}\in N_{X}(x), w_{i}\in I(X)}} \bigl(2(n_{Y}+1)+ \bigtriangleup_{Y}+2\bigtriangleup _{X} \bigr)^{\alpha}, \end{aligned} \\ & \begin{aligned} \chi_{\alpha} \bigl(R(X)\boxdot Y \bigr)&\leq2^{\alpha}m_{X}(n_{Y}+2)^{\alpha } \delta_{X}^{\alpha}+2m_{X} \bigl(n_{Y}( \delta_{X}+2)+2(\delta _{Y}+1) \bigr)^{\alpha} \\ &\quad{} +2^{\alpha}n_{X}m_{Y}(\delta_{Y}+2 \delta_{X})^{\alpha} \\ &\quad{} +n_{Y}\sum_{\substack{x\in V(X),\\[-1pt]w_{i}\in N_{X}(x), w_{i}\in V(X)}} \bigl( \delta_{X}(n_{Y}+2)+\delta_{Y}+2 \delta_{X} \bigr)^{\alpha} \\ &\quad{} +n_{Y}\sum_{\substack{x\in V(X),\\[-1pt]w_{i}\in N_{X}(x), w_{i}\in I(X)}} \bigl(2(n_{Y}+1)+ \delta_{Y}+2\delta_{X} \bigr)^{\alpha}. \end{aligned} \end{aligned}$$

*The equality holds if and only if* *X* *and* *Y* *are regular graphs*.

#### Proof

Using (3.1) and (3.5) in equation (1.1), we get

3.11 $$\begin{aligned} \chi_{\alpha} \bigl(R(X)\boxdot Y \bigr)&=\sum_{xy\in E(R(X)} \bigl(d_{R(X)}(x)+d_{R(X)}(y) \bigr)^{\alpha}+n_{X}\sum_{xy\in E(Y)} \bigl(d_{Y}(x)+d_{Y}(y) \bigr)^{\alpha} \\ &\quad{} +\sum_{x\in V(R(X))}\sum_{y\in V(Y)} \bigl(d_{R(X)}(x)+d_{Y}(y) \bigr)^{\alpha} \\ &=\sum_{\substack{xy\in E(R(X)), \\x,y\in V(X)}} \bigl(d_{X}(x) (n_{Y}+2)+d_{Y}(y) (n_{Y}+2) \bigr)^{\alpha} \\ &\quad{} +\sum_{\substack{xy\in E(R(X)), \\x\in V(X),y\in I(X)}} \bigl(d_{X}(x) (n_{Y}+2)+2(n_{Y}+1) \bigr)^{\alpha} \\ &\quad{} +n_{X}\sum_{xy\in E(Y)} \bigl(d_{Y}(x)+2d_{X}(w_{i})+d_{Y}(y)+2d_{X}(w_{i}) \bigr)^{\alpha} \\ &\quad{} +\sum_{\substack{x\in V(X) \\w_{i}\in N_{X}(x), w_{i}\in V(X)}}\sum_{y\in V(Y)} \bigl(d_{X}(w_{i}) (n_{Y}+2)+d_{Y}(y)+2d_{X}(x) \bigr)^{\alpha} \\ &\quad{} +\sum_{\substack{x\in V(X), \\w_{i}\in N_{X}(x), w_{i}\in I(X)}}\sum_{y\in V(Y)} \bigl(2(n_{Y}+1)+d_{Y}(y)+2d_{X}(x) \bigr)^{\alpha } \\ &\geq2^{\alpha}m_{X}(n_{Y}+2)^{\alpha} \bigtriangleup_{X}^{\alpha }+2m_{X} \bigl(n_{Y}( \bigtriangleup_{X}+2) +2(\bigtriangleup_{Y}+1) \bigr)^{\alpha} \\ &\quad{} +2^{\alpha}n_{X}m_{Y}( \bigtriangleup_{Y}+2\bigtriangleup _{X})^{\alpha} \\ &\quad{} +n_{Y}\sum_{\substack{x\in V(X),\\w_{i}\in N_{X}(x), w_{i}\in V(X)}} \bigl( \bigtriangleup_{X}(n_{Y}+2)+\bigtriangleup_{Y}+2 \bigtriangleup _{X} \bigr)^{\alpha} \\ &\quad{} +n_{Y}\sum_{\substack{x\in V(X),\\w_{i}\in N_{X}(x), w_{i}\in I(X)}} \bigl(2(n_{Y}+1)+ \bigtriangleup_{Y}+2\bigtriangleup _{X} \bigr)^{\alpha}. \end{aligned}$$

Similarly, we can show that

3.12 $$ \begin{aligned}[b] \chi_{\alpha} \bigl(R(X)\boxdot Y \bigr)&\leq2^{\alpha}m_{X}(n_{Y}+2)^{\alpha } \delta_{G}^{\alpha} \\ &\quad{} +2m_{X} \bigl(n_{Y}( \delta_{X}+2)+2(\delta _{Y}+1) \bigr)^{\alpha} +2^{\alpha}n_{X}m_{Y}(\delta_{Y}+2 \delta_{X})^{\alpha} \\ &\quad{} +n_{Y}\sum_{\substack{x\in V(X),\\w_{i}\in N_{X}(x), w_{i}\in V(X)}} \bigl( \delta_{X}(n_{Y}+2)+\delta_{Y}+2 \delta_{X} \bigr)^{\alpha} \\ &\quad{} +n_{Y}\sum_{\substack{x\in V(X),\\w_{i}\in N_{X}(x), w_{i}\in I(X)}} \bigl(2(n_{Y}+1)+ \delta_{Y}+2\delta_{X} \bigr)^{\alpha}. \end{aligned} $$

The equality in (3.11) and (3.12) obviously holds if and only if *X* and *Y* are regular graphs. This completes the proof. □

In the following theorem, we compute lower and upper bounds on the general sum-connectivity index for *R*-edge neighborhood corona product of $R(X)$ and *Y*.

#### Theorem 3.4

*Let* $\alpha<0$. *Then the bounds for the general sum*-*connectivity index of* $R(X)\boxminus Y$ *are given by*

$$\begin{aligned}& \begin{aligned} \chi_{\alpha} \bigl(R(X)\boxminus Y \bigr)& \geq2^{\alpha}m_{X}(n_{Y}+2)^{\alpha } \bigtriangleup_{X}^{\alpha}+2m_{X} \bigl(n_{Y} \bigtriangleup_{X}+ 2(\bigtriangleup_{X}+1) \bigr)^{\alpha} +2^{\alpha}n_{X}m_{Y}( \bigtriangleup_{Y}+2)^{\alpha} \\ &\quad{} +n_{Y}\sum_{\substack{x\in I(X),\\w_{i}\in N_{X}(x), w_{i}\in V(X)}} \bigl( \bigtriangleup_{X}(n_{Y}+2)+\bigtriangleup _{Y}+2 \bigr)^{\alpha}, \end{aligned} \\& \begin{aligned} \chi_{\alpha} \bigl(R(X)\boxminus Y \bigr)&\leq2^{\alpha}m_{X}(n_{Y}+2)^{\alpha } \delta_{X}^{\alpha}+2m_{X} \bigl(n_{Y} \delta_{X}+2(\delta _{X}+1) \bigr)^{\alpha} +2^{\alpha}n_{X}m_{Y}(\delta_{Y}+2)^{\alpha} \\ &\quad{} +n_{Y}\sum_{\substack{x\in I(X),\\w_{i}\in N_{X}(x), w_{i}\in V(X)}} \bigl( \delta_{X}(n_{Y}+2)+\delta_{Y}+2 \bigr)^{\alpha}. \end{aligned} \end{aligned}$$

*The equality holds if and only if* *X* *and* *Y* *are regular graphs*.

#### Proof

Using (3.1) and (3.6) in equation (1.1), we get

3.13 $$ \begin{aligned}[b] &\chi_{\alpha} \bigl(R(X)\boxminus Y \bigr)\\ &\quad =\sum_{xy\in E(R(X)} \bigl(d_{R(X)}(x)+d_{R(X)}(y) \bigr)^{\alpha}+m_{X}\sum_{xy\in E(Y)} \bigl(d_{Y}(x)+d_{Y}(y) \bigr)^{\alpha} \\ &\quad \quad{} +\sum_{x\in V(R(X))}\sum_{y\in V(Y)} \bigl(d_{R(X)}(x)+d_{Y}(y) \bigr)^{\alpha} \\ &\quad =\sum_{\substack{xy\in E(R(X)),\\x,y\in V(X)}} \bigl(d_{X}(x) (n_{Y}+2)+d_{X}(y) (n_{Y}+2) \bigr)^{\alpha} \\ &\quad \quad{} +\sum_{\substack{xy\in E(R(X)),\\x\in V(X),y\in I(X)}} \bigl(d_{X}(x) (n_{Y}+2)+2 \bigr))^{\alpha}+n_{X}\sum _{xy\in E(Y)} \bigl(d_{Y}(x)+2+d_{Y}(y)+2 \bigr)^{\alpha} \\ &\quad \quad{} +\sum_{\substack{x\in I(X),\\w_{i}\in N_{X}(x), w_{i}\in V(X)}}\sum_{y\in V(Y)} \bigl(d_{X}(w_{i}) (n_{Y}+2)+d_{Y}(x)+2 \bigr)^{\alpha } \\ &\quad \geq2^{\alpha}m_{X}(n_{Y}+2)^{\alpha} \bigtriangleup_{X}^{\alpha }+2m_{X} \bigl(n_{Y} \bigtriangleup_{X} +2(\bigtriangleup_{X}+1) \bigr)^{\alpha} +2^{\alpha}n_{X}m_{Y}( \bigtriangleup_{Y}+2)^{\alpha} \\ &\quad \quad{} +n_{Y}\sum_{\substack{x\in I(X),\\w_{i}\in N_{X}(x), w_{i}\in V(X)}} \bigl( \bigtriangleup_{X}(n_{Y}+2)+\bigtriangleup _{Y}+2 \bigr)^{\alpha}. \end{aligned} $$

Analogously, one can compute the upper bound,

3.14 $$\begin{aligned} \chi_{\alpha} \bigl(R(X)\boxminus Y \bigr)&\leq2^{\alpha}m_{X}(n_{Y}+2)^{\alpha } \delta_{X}^{\alpha}+2m_{X} \bigl(n_{Y} \delta_{X}+2(\delta _{X}+1) \bigr)^{\alpha} +2^{\alpha}n_{X}m_{Y}(\delta_{Y}+2)^{\alpha} \\ &\quad{} +n_{Y}\sum_{\substack{x\in I(X),\\w_{i}\in N_{X}(x), w_{i}\in V(X)}} \bigl( \delta_{X}(n_{Y}+2)+\delta_{Y}+2 \bigr)^{\alpha}. \end{aligned}$$

The equality in (3.13) and (3.14) obviously holds if and only if *X* and *Y* are regular graphs. This completes the proof. □

### Line graph

The line graph of *X*, denoted by $L(X)$, is a graph with vertex set $V(L(X))=E(X)$ and any two vertices $e_{1}$ and $e_{2}$ have an arc in $L(X)$ if and only if they share a common endpoint in *X*. The graph $L(X)$ has $m_{X}$ vertices and $\frac{1}{2}M_{1}(X)-m_{X}$ edges. The degree of a vertex $x\in L(X)$ is given by

3.15 $$ d_{L(X)}(x)=d_{X}(w_{i})+d_{X}(w_{j})-2 \quad\mbox{if } x=w_{i}w_{j}, \forall w_{i},w_{j} \in V(X). $$

We compute lower and upper bounds on the general sum-connectivity index of $L(X)$ in the following theorem.

#### Theorem 3.5

*Let* $\alpha<0$. *Then the bounds for the general sum*-*connectivity index of* $L(X)$ *are given by*

$$4^{\alpha}(\bigtriangleup_{X}-1)^{\alpha} \biggl( \frac {1}{2}M_{1}(X)-m_{X} \biggr)\leq \chi_{\alpha} \bigl(L(X) \bigr)\leq4^{\alpha}(\delta_{X}-1)^{\alpha} \biggl(\frac{1}{2}M_{1}(X)-m_{X} \biggr). $$

*The equality holds if and only if* *X* *is a regular graph*.

#### Proof

Using (3.1) and (3.15) in equation (1.1), we get

3.16 $$ \begin{aligned}[b] \chi_{\alpha} \bigl(L(X) \bigr)&= \sum_{xy\in E(L(X)} \bigl(d_{L(X)}(x)+d_{L(X)}(y) \bigr)^{\alpha} \\ &=\sum_{\substack{x=w_{i}w_{j} \in E(X),y=w_{j}w_{k}}} \bigl(d_{X}(w_{i})+d_{X}(w_{j})-2+d_{X}(w_{j})+d_{X}(w_{k})-2 \bigr) \\ &\geq4^{\alpha}(\bigtriangleup_{X}-1)^{\alpha} \biggl( \frac {1}{2}M_{1}(X)-m_{X} \biggr). \end{aligned} $$

One can analogously compute the following:

3.17 $$ \chi_{\alpha} \bigl(L(X) \bigr)\leq4^{\alpha}( \delta_{X}-1)^{\alpha} \biggl(\frac{1}{2}M_{1}(X)-m_{X} \biggr). $$

The equality in (3.16) and (3.17) holds if and only if *X* is a regular graph. □

### Rooted product

A rooted graph is graph in which one vertex is labeled as a special vertex and that vertex is called root vertex of graph. The rooted graph is also known as a pointed graph and a flow graph. Let *Y* be a labeled graph with $n_{Y}$ and *X* be a sequence of $n_{Y}$ rooted graphs $X_{1},X_{2},\ldots,X_{n_{Y}}$. The rooted product of *X* and *Y*, denoted by $Y(X)$, is a graph that obtained from one copy of *Y* and $n_{Y}$ copies of *X* and identifying the rooted vertex of $X_{i}$ ($1\leq i\leq n_{Y}$) with *i*th vertex of *Y*. The number of vertices and edges in $Y(X)$ are $n_{X}=n_{X_{1}}+n_{X_{2}}+\cdots+n_{X_{n_{Y}}}$ and $m_{X}+m_{Y}$. The degree of a vertex $x\in V(Y(X))$ (where $w_{i}$ is a rooted vertex of $X_{i}$) is given by

3.18 $$ d_{Y(X)}(x)= \textstyle\begin{cases} d_{Y}(x)+d_{X_{i}}(w_{i}) &\mbox{if }x\in V(Y), \\ d_{Y}(x)+d_{X_{i}}(w_{i}) &\mbox{if }x\in V(X),x=w_{i},\\ d_{X_{i}}(x) &\mbox{if }x\in V(X),x\neq w_{i}. \end{cases} $$

In the following theorem, the bounds on the general sum-connectivity index of rooted product are computed.

#### Theorem 3.6

*Let* $\alpha<0$. *Then the bounds for the general sum*-*connectivity index of* $Y(X)$ *are given by*

$$\begin{aligned}& \begin{aligned} \chi_{\alpha} \bigl(Y(X) \bigr)&\geq\sum _{ij\in E(Y)}(2\bigtriangleup _{Y}+\omega_{i}+ \omega_{j})^{\alpha}+ \sum_{i=1}^{n_{Y}} \chi_{\alpha}(X_{i})\\ &\quad{} +\sum_{i=1}^{n_{Y}} \bigl\vert N_{X_{i}}(w_{i}) \bigr\vert \bigl[(2 \bigtriangleup_{X_{i}} +\bigtriangleup_{Y})^{\alpha}-2^{\alpha} \bigtriangleup _{X_{i}}^{\alpha} \bigr], \end{aligned} \\& \chi_{\alpha} \bigl(Y(X) \bigr)\leq\sum_{ij\in E(Y)}(2 \delta _{Y}+\omega_{i}+\omega_{j})^{\alpha}+ \sum_{i=1}^{n_{Y}}\chi_{\alpha}(X_{i})+ \sum_{i=1}^{n_{Y}} \bigl\vert N_{X_{i}}(w_{i}) \bigr\vert \bigl[(2\delta_{X_{i}} + \delta_{Y})^{\alpha}-2^{\alpha}\delta_{X_{i}}^{\alpha} \bigr]. \end{aligned}$$

*The equality holds if and only if* *Y* *and* $X_{i}$ *are regular graphs*.

#### Proof

Let the degree of $w_{i}$ in $X_{i}$ be denoted by $\omega_{i}$ and the number of neighbors of $w_{i}$ in $X_{i}$ be denoted by $\vert N_{X_{i}}(w_{i}) \vert $. Using (3.1) and (3.18) in equation (1.1), we get

$$\begin{aligned} &\chi_{\alpha} \bigl(Y(X) \bigr) \\ &\quad =\sum _{ij\in E(Y)} \bigl(d_{Y}(i)+\omega _{i}+d_{Y}(j)+ \omega_{j} \bigr)^{\alpha} \\ &\quad \quad{} +\sum_{i=1}^{n_{Y}} \biggl[\sum _{\substack{xy\in E(X_{i}),\\x,y\neq w_{i}}} \bigl(d_{X_{i}}(x)+d_{X_{i}}(y) \bigr)^{\alpha} +\sum_{\substack{xy\in E(X_{i}),\\x\in V(X_{i}),y=w_{i}}} \bigl(d_{X_{i}}(x)+d_{Y}(i)+ \omega_{i} \bigr)^{\alpha} \biggr] \\ &\quad =\sum_{ij\in E(Y)} \bigl(d_{Y}(i)+ \omega_{i}+d_{Y}(j)+\omega _{j} \bigr)^{\alpha} \\ &\quad \quad{} +\sum_{i=1}^{n_{Y}} \biggl[ \chi_{\alpha}(X_{i})-\sum_{\substack{xy\in E(X_{i}),\\x\in V(X_{i}),y=w_{i}}} \bigl(d_{X_{i}}(x)+\omega _{i} \bigr)^{\alpha}+\sum _{\substack{xy\in E(X_{i}),\\x\in V(X_{i}),y=w_{i}}} \bigl(d_{X_{i}}(x)+d_{Y}(i)+ \omega_{i} \bigr)^{\alpha} \biggr] \\ &\quad \geq\sum_{ij\in E(Y)}(2\bigtriangleup_{Y}+ \omega_{i}+\omega _{j})^{\alpha}+ \sum _{i=1}^{n_{Y}}\chi_{\alpha}(X_{i})+\sum _{i=1}^{n_{Y}} \bigl\vert N_{X_{i}}(w_{i}) \bigr\vert \bigl[(2\bigtriangleup_{X_{i}} +\bigtriangleup_{Y})^{\alpha}-2^{\alpha} \bigtriangleup _{X_{i}}^{\alpha} \bigr]. \end{aligned}$$

Similarly, we can show that

$$ \chi_{\alpha} \bigl(Y(X) \bigr)\leq\sum _{ij\in E(Y)}(2\delta _{Y}+\omega_{i}+ \omega_{j})^{\alpha}+ \sum_{i=1}^{n_{Y}} \chi_{\alpha}(X_{i})+\sum_{i=1}^{n_{Y}} \bigl\vert N_{X_{i}}(w_{i}) \bigr\vert \bigl[(2 \delta_{X_{i}} +\delta_{Y})^{\alpha}-2^{\alpha} \delta_{X_{i}}^{\alpha} \bigr]. $$

The equality in (3.13) and (3.14) obviously holds if and only if *Y* and $X_{i}$ are regular graphs. This completes the proof. □

In the special case, when all $X_{1},X_{2},X_{3},\ldots,X_{n_{Y}}$ are isomorphic to a graph *G*, then the rooted product of *Y* and *G* is denoted by $Y\{G\}$. This rooted product is called a cluster of *Y* and *G*. The following corollary is an easy consequence of Theorem 3.6.

#### Corollary 3.1

*Let* $\alpha<0$. *Then the bounds for the general sum*-*connectivity index of cluster* $Y\{G\}$ *are given by*

$$\begin{aligned}& \chi_{\alpha} \bigl(Y\{G\} \bigr) \geq2^{\alpha}m_{Y}(\bigtriangleup _{Y}+ \omega)^{\alpha}+n_{Y}\chi_{\alpha}(G) +n_{Y} \bigl\vert N_{G}(w) \bigr\vert \bigl[(2\bigtriangleup _{G}+\bigtriangleup_{Y})^{\alpha}-2^{\alpha} \bigtriangleup _{G}^{\alpha} \bigr], \\& \chi_{\alpha} \bigl(Y\{G\} \bigr)\leq2^{\alpha}m_{Y}( \delta_{Y}+\omega )^{\alpha}+n_{Y}\chi_{\alpha}(G) +n_{Y} \bigl\vert N_{G}(w) \bigr\vert \bigl[(2 \delta_{G}+\delta _{Y})^{\alpha}-2^{\alpha} \delta_{G}^{\alpha} \bigr], \end{aligned}$$

*where* $\omega=d_{G}(w)$ *and* $\vert E(G) \vert =m_{Y}$.

## Conclusion

In this article, we obtained the lower and upper bounds for the general sum-connectivity index of four types of graph operations involving *R*-graph. Additionally, we have determined the bounds for the general sum-connectivity index of line graph $L(X)$ and the rooted product of graphs.
